# L-Ascorbic Acid as an Efficient Green Corrosion Inhibitor of Steel Rebars in Chloride Contaminated Cement Mortar

**DOI:** 10.3390/ma15228005

**Published:** 2022-11-12

**Authors:** Cristina Argiz, Celia Arroyo, Astrid Bravo, Amparo Moragues, Carmen Andrade, Fabio Bolzoni

**Affiliations:** 1 E.T.S.I. Caminos, Canales y Puertos, Universidad Politécnica de Madrid, 20040 Madrid, Spain; 2 Centro Internacional de Métodos Numéricos en Ingeniería (CIMNE), 08034 Madrid, Spain; 3 Politecnico di Milano, 20133 Milano, Italy

**Keywords:** green corrosion inhibitor, ascorbic acid, chloride-contaminated mortars, linear polarization resistance, electrochemical impedance spectroscopy, differential thermal, thermogravimetric analysis

## Abstract

Corrosion of reinforcement is a major problem regarding concrete durability. In new structures the corrosion onset can be delayed if additional protection methods are provided as is the case for the addition of corrosion inhibitors in the concrete mix. The main goal of this paper is the evaluation of the effect of the ascorbic acid (AA) as a green steel corrosion inhibitor in cement mortars contaminated by chlorides. Concentration levels of ascorbic acid, ranging from 0.5 to 10^−3^ mol/L, were added to the mixing water. Electrochemical methods, including corrosion potential (Ecorr), linear polarization resistance (LPR) and electrochemical impedance spectroscopy (EIS), were employed to assess the corrosion rate of the steel embedded in the mortars. The corrosion inhibiting performance of ascorbic acid was compared with that of sodium nitrite. The interaction of the ascorbic acid with the hydrated cement matrix was also evaluated with differential thermal and thermogravimetric analysis (DTA/TG) and pH measurements. The results indicated that, depending on the ascorbic acid concentration, it can be an activator of the corrosion process or an effective corrosion inhibitor in a similar manner to sodium nitrite. A corrosion rate decrease was achieved with concentrations below 10^−2^ mol/L and the optimum content was 10^−3^ mol/L. Within this concentration range, the AA does not modify the hydration performance of the cement matrix.

## 1. Introduction

Corrosion of reinforced concrete steel due to the presence of chloride ions is one of the main reasons for the deterioration of reinforced concrete structures. The steel reinforcement in concrete is normally passivated due to the high alkalinity of the concrete. The protective passive film on the steel surface is composed of iron oxides and oxy-hydroxides. The critical chloride content or threshold value (Ccrit) is commonly expressed as total chloride content relative to the weight of the cement [1]. Considering that only free chloride presents a corrosion risk, the threshold level can be reported as free chloride or the chloride to hydroxyl concentration ratio in the pore solution (Cl^−^)/(OH^−^) [2]. In terms of the total chloride added in the mixing water, Alonso et al. [3] found that it ranges between 1.24 and 3.08% by weight of cement and between 1.17 and 3.98 considering the [Cl^−^]/[OH^−^] molar ratio for chlorides admixed in the mixing water.

The use of corrosion inhibitors in reinforced concrete structures is one of the most efficient methods for reducing and preventing corrosion of concrete steel reinforcements due to its ease of application, i.e., it is added to the mixing water, is compatible with the concrete and has relatively low cost. Nitrites (calcium or sodium salts) are the most known and longest used anodic corrosion inhibitor in concrete [4,5]. According to Gaidis [6,7], nitrite oxidizes ferrous iron (Fe^2+^) to generate ferric iron (Fe^3+^), leading to the formation of a passivating layer, while the nitrite form is reduced to nitric oxide.

The corrosion inhibitor performance of the nitrite ions is guaranteed only when the molar ratio of nitrite ions to chloride ions is higher than a certain value. Different authors have reported the significance of this critical concentration ratio, [Cl^−^]/[NO_2_^−^], although with different values. Gaidis and Rosenberg indicated that [Cl^−^]/[NO_2_^−^] ratios not exceeding 1.5 can provide passivity [7]. Elsener [8] said that to prevent chloride induced corrosion, the optimum nitrite to chloride ratio is in the range of 0.8 to 1.0. According to Ormellese [9], the chloride level of corrosion initiation corresponds to a [Cl^−^]/[NO_2_^−^] molar ratio within the range of 1.1 to 2.0. Ann et al. [10] investigated the influence of calcium nitrite-based corrosion inhibitors added to the mixing water on the chloride transport. Their results showed a [NO_2_^−^]/[Cl^−^] threshold level ranging from 0.34 to 0.66 to prevent corrosion.

However, the application of the nitrite inhibitors in corrosion protection is limited due to their toxicity and carcinogenicity. Sustainability requirements demand the use of non-hazardous, low-cost, and environmentally friendly products, which are known as “green” or biocompatible inhibitors. Most of green inhibitors are heterocyclic compounds containing one or more heteroatoms, such as N, O, P, and S, with lone pairs and aromatic rings with π-electrons that can undergo adsorption with the metal surface [11]. The inhibiting action of those organic compounds is usually attributed to interactions with metallic surfaces by adsorption.

Vitamin C or L-ascorbic acid (C_6_H_8_O_6_) (AA) is a hydrosoluble weak dibasic acid, a strong antioxidant and a free-radical scavenger. Its molecular structure is a five-membered heterocyclic lactone ring with an enediol group at the carbons C_2_ and C_3_. These two acidic protons are the reason for the acidic properties of the molecule. The C_3_ hydroxyl proton conjugated with the C_1_ carbonyl group results in significant acidity (pK_1_ = 4.2) as compared to the C_2_ hydroxyl group (pK_2_ = 11.6) [12]. Ascorbic acid oxidizes reversibly to L-dehydroascorbic acid (DHA), involving the transfer of two electrons and two protons [13]. DHA has antioxidant properties of its own. In neutral solution, it is unstable and rapidly hydrolyzed to 2,3-diketo-Lgulonic acid (DKA). DKA is an oxygen-accepting antioxidant and appears to be a more potent reducing agent than AA. It is a highly unstable compound and further degrades to over 50 different products [14,15,16,17,18].

In terms of physiological conditions, when pH is between pK_1_ and pK_2_, the predominant specie is the ascorbate monoanion (AH^−^) which is oxidized with one-electron transfer to the ascorbate radical (A^•−^) and a second one-electron transfer to give dehydroascorbic acid (DHA). A pH > pK_2_ ascorbate radical (A^•−^) is formed by one electron oxidation of A^2−^ without any proton transfer. The bicyclic hydrated form of dehydroascorbic acid (DHA) was found to be the most stable structure of the fully oxidized form of ascorbic acid in aqueous solution [19,20].

A review of the literature revealed that there have been few reports on the effect of ascorbic acid as a corrosion inhibitor, and most of them have been reported for acid solutions. The influence of L-ascorbic acid (H_2_A) on mild steel corrosion in acidic solutions (H_2_SO_4_ 0.01 M, pH = 2–6) was studied by Ferreira et al. [21]. AA concentrations between 10^−3^ and 10^−7^ mol/L were studied. The results obtained by using potentiodynamic polarization curves and weight loss measurements showed that a maximum inhibition efficiency of about 69% could be achieved in the presence of 10^−3^ mol/L of ascorbic acid in pH = 4 solutions. The inhibitory effect of L-ascorbic acid on the corrosion process of low carbon steel in 0.5 M Na_2_SO_4_ solutions was also confirmed by Gonçalves and Mello [22].

Anejjaret al. [23] studied the effect of vitamin C as an inhibitor with concentrations between 10^−2^ and 10^−5^ mol/L in 1 M HCl solutions. The results revealed the effect of AA as a corrosion inhibitor in acidic solutions. Its effectiveness is enhanced when the inhibitor concentration increases. The highest efficiency (87.62%) is achieved with 10^−2^ M of ascorbic acid. 

The corrosion inhibition of ascorbic acid in 0.03% NaCl solutions was studied by Sekine et al. [24], showing a maximum corrosion inhibition with a concentration of 200 ppm (10^−3^ mol/L) at pH = 6. It was determined that ascorbic acid is adsorbed on the surface of the metal. The inhibitory effect is reduced at high concentrations due to the formation of Fe(II) chelates.

Akrout et al. [25] analyzed the performance of L-ascorbic acid with concentrations ranging from 0.057 to 1.703 mmol/L, in solutions with 2 × 10^−2^ M NaCl and pH = 8. The presence of the inhibitor on the steel surface was confirmed by Raman spectroscopy. In general, the polarization resistance Rp increases as the inhibitor concentration increases. There was some evidence that the ascorbic acid acts by adsorption on the steel surface.

In a highly alkaline medium, the study undertaken by Valek, et al. [26] in saturated Ca(OH)_2_ solutions with 0.14 mol/L of chloride containing several AA concentrations (10^−2^, 10^−3^ and 10^−4^ mol/L) showed that the concentration of 10^−3^ mol/L AA attains the maximum inhibition with a charge transfer resistance Rct = 166.3 KΩ·cm^2^ (icorr = 0.16 µA/cm^2^) three times higher compared to that in the blank solution, Rct = 58.1 KΩ·cm^2^ (icorr = 0.45 µA/cm^2^). The inhibition effect decreased at higher concentrations due to the formation of iron chelates.

The aim of the present study is the evaluation in mortar specimens of ascorbic acid as a potential green corrosion inhibitor compared to an inorganic well-known and effective inhibitor, sodium nitrite. Electrochemical measurements of the corrosion potential, linear polarization resistance, electrochemical impedance spectroscopy (EIS) and weight loss were carried out in mortars specimen long-term. Furthermore, characterization analysis to evaluate the interaction of the ascorbic acid with the hydrated cement matrix was performed with differential thermal and thermogravimetric analysis (DTA/TG) and pH measurements.

## 2. Materials and Methods

### 2.1. Materials

A common Portland cement CEM I 52.5R-SR3 (according to the European standard EN197-1:2011) supplied by Carboneras cement plant, Holcim, Spain, Blaine SSA of 5385 cm^2^/g, was used to produce mortars and pastes. The chemical composition is given in Table 1. Two reinforcing steel corrosion inhibitors were tested: L(+)-ascorbic acid (AA) and NaNO_2_, with a purity > 99%, provided by Labkem.

Mortar specimens, made for electrochemical measurements, were cast in prismatic molds with dimensions of 8 × 5.5 × 2 cm^3^ as used elsewhere [3]. Mortars were made according to EN 196-1:2016, with a cement/sand/water ratio of 1/3/0.5. Two corrugated reinforcing steel bars of 6 mm in diameter by 90 mm length (B500SD, UNE 36068: 2011) and chemical composition (wt.%) 0.30 C, 0.29 Si, 0.40 Mn, 0.05 S, 0.05 z and balance of Fe to 100% were embedded in the mortar specimens as working electrodes, a graphite bar placed in the middle of the two working electrodes was used as a counter electrode. The steel bars were previously cleaned in a ultrasound bath, immersed in a HCl:H_2_O (1:1) solution with hexamethylentetramine (urotropine) 3.5 g/L, washed in tap water and deionized water and air dried. An adhesive insulating tape was used, limiting an exposed area of 5.65 cm^2^. The specimens were cured in a humid chamber at 20 °C and 98% RH for 24 h until demolding. Later on, they were kept in the same humid chamber until testing time.

Cement pastes were manufactured to evaluate the effect of ascorbic acid on the cement paste matrix without addition of chloride ions. Cement paste specimens were pre-pared in 6 × 1 × 1 cm^3^ molds with a water/cement ratio of 0.35. Cement pastes were cured for 2, 7 and 28 days in the same conditions than mortars. The hydration was stopped at the testing times by holding the samples in a vacuum chamber for 30 min then by immersion in isopropanol for two hours, and finally they were kept in an oven at 40 °C until characterization analysis. The concentration of ascorbic acid added in the mixing water of the mortar specimens and cement pastes is given in Table 2.

Furthermore, to compare de inhibition effect of ascorbic acid with that of the nitrite ions, a mortar specimen was prepared. The amount of NaNO_2_ added to the mixing water was 3.89% of NaNO_2_ by weight of cement, corresponding to 2.6% of NO_2_^−^ ions by weight of cement or a concentration of 1.13 mol/L of NO_2_^−^ ions in the mixing water.

For all mortars, chloride ions were added to the mixing water, 3.29% of NaCl by weight of cement, which is equivalent to 2% of Cl^−^ ions by weight of cement or to a concentration of 1.13 mol/L of Cl^−^ ions added to the mixing water. The [Cl^−^]/[NO_2_^−^] molar ratio was 1.

### 2.2. Methods

The electrochemical measurements were performed in the mortars specimens using a potentiostat/galvanostat with FRA 32 module Autolab PGSTAT 204 provided by Metrohm, Spain, to measure the electrochemical impedance spectroscopy (EIS) using Nova 2.4.1 software package. The potential was measured through a 3 M KCl Ag/AgCl (Metrohm) reference electrode. The open circuit potential (OCP) was first recorded for 300 s or until the potential change with time dV/dt was ≤10^−6^ V/s. Linear polarization resistance Rp values were then obtained from the slope ∆E/∆I applying ±20 mV at a scan rate of 0.1667 mV/s, at the corrosion potential. The corrosion current density (Icorr) was calculated from the Stern and Geary equation: Icorr = B/Rp [27] adopting a value of 26 mV for the B constant. The electrochemical impedance spectroscopy (EIS measurements were recorded with a logarithmic frequency swept from 100 KHz to 0.01 Hz acquiring 10 points per decade and an amplitude of 10^−2^ V.

In the hydrated cement pastes, differential thermal and thermogravimetric analysis (DTA-TG), using a LABSYS EVO equipment provided by Setaram Instrumentation, Spain, was performed by heating around 60 mg of sample and applying a dynamic ramp from 40 to 1100 °C with a heating rate of 10 °C/min in N_2_ atmosphere in crucibles made of alumina (α-Al_2_O_3_) as reference material.

The pH measurements were taken with a CRISON pH meter with temperature compensation and calibrated with three standard buffer solutions. The measurement method is based on the ex situ leaching (ESL) procedure [28]. This is a frequently used method for measuring the pH of soil (ASTM D4972-13 [29]). The method consists of weighing 10 g of grounded cement paste and mixing it with 10 mL of deionized CO_2_-free water and stirring continuously for 5 min using a magnetic stirrer. The pH was measured, introducing the pH electrode into the suspension without filtering.

## 3. Results

### 3.1. Electrochemical Measurements in Mortars

#### 3.1.1. Open Circuit Potential and Linear Polarization Resistance (LPR)

**Figure 1:**
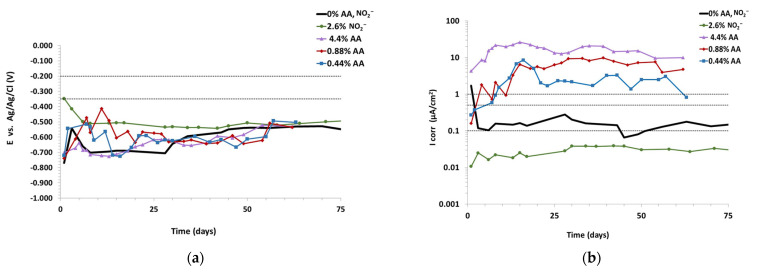
Chloride-contaminated mortars with 4.4, 0.88 and 0.44% of AA and 2.6% of NO_2_^−^ (**a**) OCP, (**b**) Icorr.

The open circuit potential (OCP) data of the chloride-contaminated mortars with high ascorbic acid amounts (4.4, 0.88 and 0.44% of AA) by weight of cement and a mortar with nitrite, 2.6% of NO_2_^−^ by weight of cement, were recorded periodically up to 75 days. Results are depicted in Figure 1a. Based on standard ASTMC876-15 [30], all the specimens shown in Figure 1a present potentials around −700 to −500 mV which would indicate that the reinforcement is actively corroding. The less negative (noble) corrosion potential corresponds to the mortar with nitrite in which the rebar maintains a corrosion potential of −500 mV during the testing time.

The evolution of corrosion current density (Icorr) vs. time, calculated from Rp measurements, is displayed in Figure 1b. The ranges of corrosion current related to the significance in terms of service life of the reinforcement were defined based on the RILEM TC 154-EMC Recommendations [31]. It can be seen that only the mortar with nitrite presents Icorr values below 0.1 µA/cm^2^. The reference mortar shows a low corrosion level between 0.1 and 0.2 µA/cm^2^. Conversely, high values of corrosion were observed for mortars with ascorbic acid inhibitor ranging from 1.5 to 25 µA/cm^2^, indicating active corrosion of the steel bars. The higher the ascorbic acid content, the higher the corrosion current density achieved.

**Figure 2:**
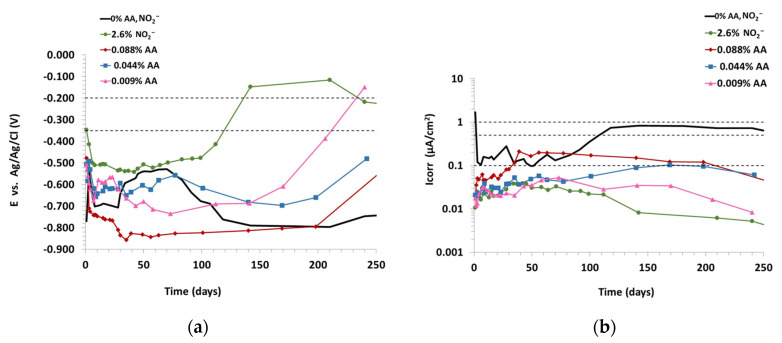
Effect of AA and NO_2_^−^ in chloride-contaminated (**a**) OCP; (**b**) Icorr.

Based on these preliminary results and the literature data in the solution test reported by Valek [26] and the degradation chemistry of ascorbic acid, it was considered to reduce the ascorbic acid content in the mixing water and to assess its long-term inhibitory effect. Figure 2a shows the results of the corrosion potential of the chloride-contaminated mortars made with the lowest amounts of ascorbic acid, 0.088, 0.044 and 0.009% of AA by weight of cement, a reference mortar with chlorides and another with 2.6% of NO_2_^−^ by weight of cement and chlorides, up to 240 days. It can be seen that mortars maintain from the beginning similar or even more negative potential values (−500 and −850 mV) than mortars with higher amounts of ascorbic acid. However, after 100 days, the potential of the mortar with nitrite and 0.009% of AA shift towards more positive values (−200 mV) which would mean a passivation of the rebars. This change requires a longer period of time for the mortars with 0.044 and 0.088% of AA and such noble potentials are not reached.

Figure 2b shows that the corrosion current density of the reference mortar with chlorides initially starts above 0.1 µA/cm^2^, then increases beyond 0.5 µA/cm^2^, indicating high risk of corrosion. The addition of low amounts of ascorbic acid markedly reduces the corrosion of reference mortar. The larger amount, 0.088% of AA, reduces the corrosion current density to values between 0.2 and 0.1 µA/cm^2^. The passive state of reinforced steel rebars Icorr < 0.1 µA/cm^2^ was reached from 0.044% of AA content. The mortars with 0.009% of AA and nitrite decrease the corrosion rate to a range between 0.05 and 0.005 µA/cm^2^. The lower the ascorbic acid content, the greater the inhibitory efficiency of AA. The lower ascorbic acid content shows similar inhibitory effectiveness to nitrite against chloride induced corrosion. The results shown in Figure 1 and Figure 2 are the average of measurements made on the two steel bars embedded in the mortar specimens.

The inhibitor efficiency *η*(%) was calculated according to the Equation (1), where *Icorr_Ref_* and *Icorr_Inh_* correspond to the integral under the corrosion current density of mortars without and with inhibitor.

(1) $$\eta \left(\%\right)=\frac{Icorr_{Ref}-Icorr_{Inh}}{Icorr_{Ref}}\cdot 100$$

Consistent with the above observations, the efficiency of the inhibitors in chloride-contaminated mortars rises with the decrease in ascorbic acid content. In Table 3, it can be seen that the inhibitory efficiency of the lower AA 97.3% (0.009% of AA) was similar to the nitrite inhibitory effectiveness 97.8% for 2.6% of nitrite.

**Figure 3:**
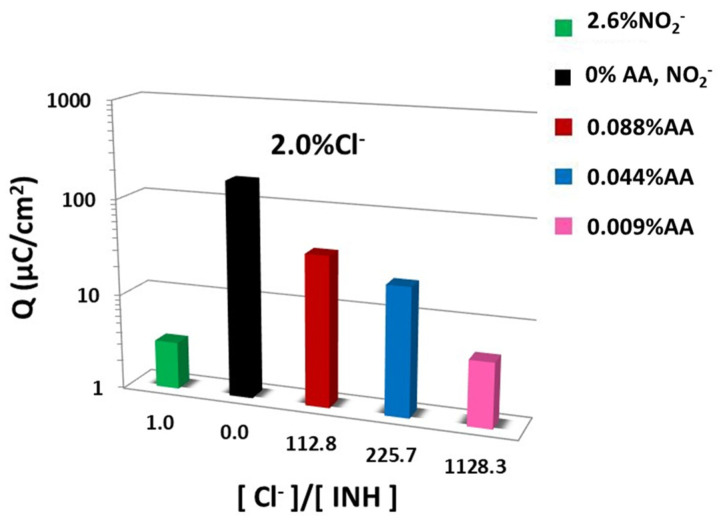
Electric charge vs. molar chloride/inhibitor concentration.

Figure 3 represents the area under the Icorr vs. time curve during the 240 testing days (electric charge/day·cm^2^). The accumulated corrosion current density decreases sharply from the reference value of 173 to 7.13 µA/cm^2^ of AA inhibitor (0.009%) and 3.13 µA/cm^2^ of nitrite inhibitor. It is important to emphasize that such a corrosion rate reduction by nitrites and ascorbic acid is achieved with a 1:1000 chloride/inhibitor molar ratio, [Cl^−^]/[NO_2_^−^] = 1 and [Cl^−^]/[AA] = 1129.

**Figure 4:**
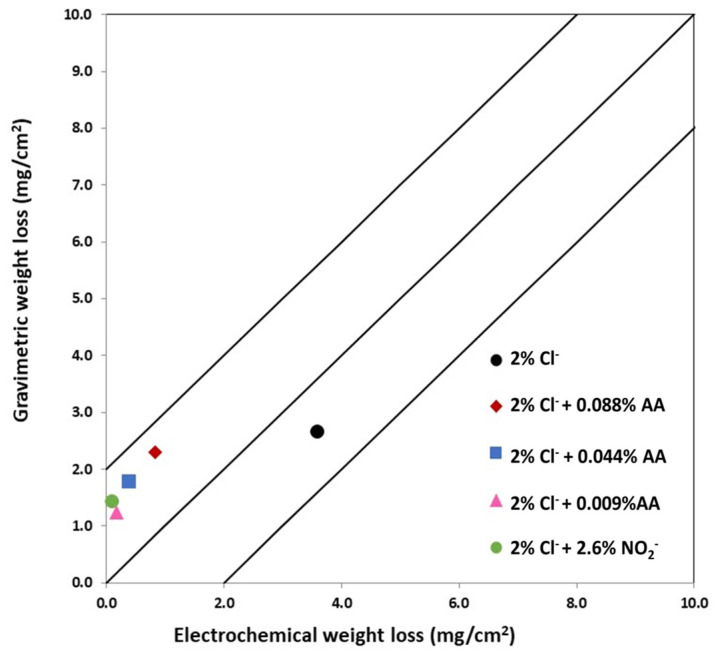
Gravimetric weight loss vs. electrochemical weight loss for chloride-contaminated mortars with inhibitors.

The gravimetric weight loss measured on steel bars after 240 days and the electro-chemical weight loss, obtained by means of the Faraday law, from the integration of the corrosion rate values, are compared in Figure 4. The central line of the graph represents the equivalence between both measurements and the two parallel lines the difference between applying B = 13 mV or a B = 52 mV [32,33]. The electrochemical weight loss of steels embedded in mortars with inhibitor was lower than gravimetric loss, probably due to the application of a B = 26 mV constant instead of B = 52 mv expected for passive systems.

However, for the steel embedded in the mortar made with chloride ions but without inhibitor, the electrochemical loss is higher, since in this case the B value used is characteristic for the steel in the active corrosion state [32]. The weight losses are lower for the steels embedded in mortars made with nitrite ions and the lowest amount of AA, growing in increasing order with the increase in the inhibitor content.

#### 3.1.2. Electrochemical Impedance Spectroscopy (EIS)

**Figure 5:**
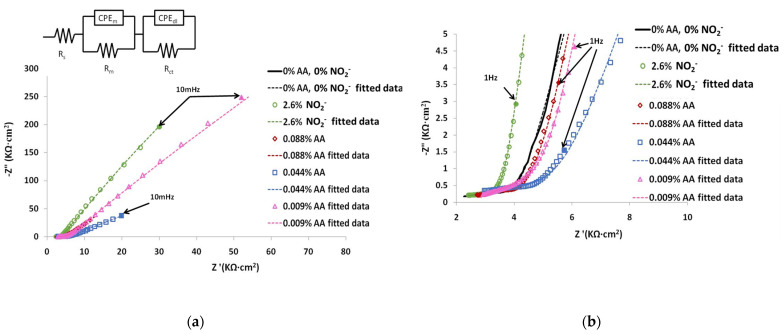
(**a**) Nyquist plots of chloride-contaminated mortars, 2% of Cl^−^ with 0.088, 0.044 and 0.009% of AA and 2.6% of NO_2_^−^ and equivalent circuit; (**b**) amplified view.

The Nyquist diagrams for the mortars with 0.088, 0.044 and 0.009% AA and 2.6% nitrites were recorded after 240 days and are shown in Figure 5a with an amplified view in Figure 5b. The Nyquist plot in Figure 5 exhibits two apparent capacitive loops with a significantly larger diameter on mortars with the addition of nitrite and 0.009% of AA inhibitors. The diameter of reference mortar was smaller than any of the mortars with inhibitors. The capacitive arc of mortars with ascorbic acid increase with the decrease in AA content. These results are consistent with LPR studies previously presented. In general, the diameter of the low-frequency capacitive arc in the Nyquist plots should be approximately equal to the value of the polarization resistance (Rp).

**Figure 6:**
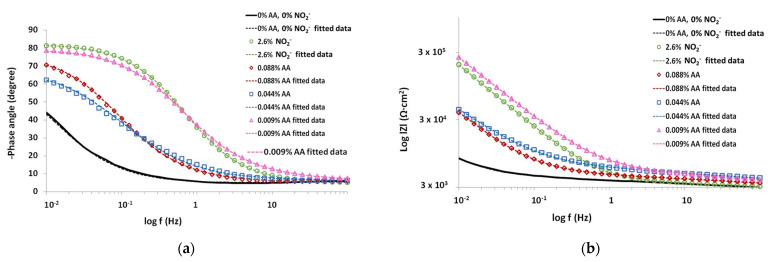
Bode plots of chloride-contaminated mortars, 2% of Cl^−^ with 0.088, 0.044 and 0.009% of AA and 2.6% of NO_2_^−^ (**a**) logarithmic frequency vs. phase angle; (**b**) logarithmic impedance modulus vs. log frequency.

From the logarithmic frequency vs. phase angle Figure 6a and logarithmic impedance modulus vs. log frequency Figure 6b of Bode plots, it is noticed that the minimum of phase angle value, around −80°, appears at low frequencies around 0.01 Hz for mortars with nitrite and 0.009% of AA inhibitors revealing the protective properties of the film. The phase angle increases at −73° for mortars with 0.088% AA and at −66° for mortars with 0.044% AA shifting towards lower frequencies of 0.005 and 0.003 Hz, respectively. The reference mortar does not reach its minimum phase angle even at frequencies of 0.001 Hz.

The EIS measurements were interpreted, fitting the impedance data to the electrical equivalent circuit insert in Figure 5 with Autolab software. This equivalent circuit has been used before for passive steel in mortars or uniform corrosion [34,35,36,37]. Dashed lines in EIS diagrams represent the fitting values using the equivalent electrical circuit. The fitting parameters are presented in Table 4.

The measured resistance parameter Rs corresponds to the electrolytic resistance of pore solution, and it was added to the Rm value in Table 4. The Rm-CPEm couple, predominating at high frequencies, may be associated with the dielectric properties of the mortar cover together with degradation products of the ascorbic acid in the mortar matrix and reflect the ability of mortar/inhibitor to resist the penetration of electrolytes containing chloride ions depending on its microstructure [36].

The (Rct–CPEdl) time constant was associated with the charge transfer resistance and double-layer capacitance of the rebar/electrolyte interface associated with the corrosion reactions on the interface between the steel surface and cover [38,39]. In the electrical equivalent circuit, the constant phase element (CPE) is in place of a pure capacitor as it is composed of the capacitance and deviation parameter due to a non-uniform thickness of the corrosion inhibitor layer, non-uniform corrosion reaction on the surface or non-uniform current distribution and surface roughness [40,41]. The impedance of the constant-phase element (CPE) can be expressed by Equation (2):

(2) ZCPE=1[Y0(jω)n]

where ZCPE is the impedance of CPE, Υ_0_ is the CPE constant (admittance magnitude of CPE), with units of Ω^−1^·cm^−2^·s^n^, j is the imaginary unit j^2^ = (−1), and ω is the angular frequency (ω = 2πf), where f represents the frequency at which the imaginary value reaches a maximum on the Nyquist plot and n is the deviation parameter of the capacitance of the electrode from the ideal condition of a pure capacitor in the range of −1 ≤ n ≤ 1. When n = 1, the CPE becomes equivalent to an ideal capacitor, and when n = 0, the CPE becomes equivalent to a resistor. When n = −1 the CPE becomes equivalent to an ideal inductor and finally, if n = 0.5, the CPE is the Warburg admittance.

### 3.2. Visual and Optical Microscope Examination

**Figure 7:**
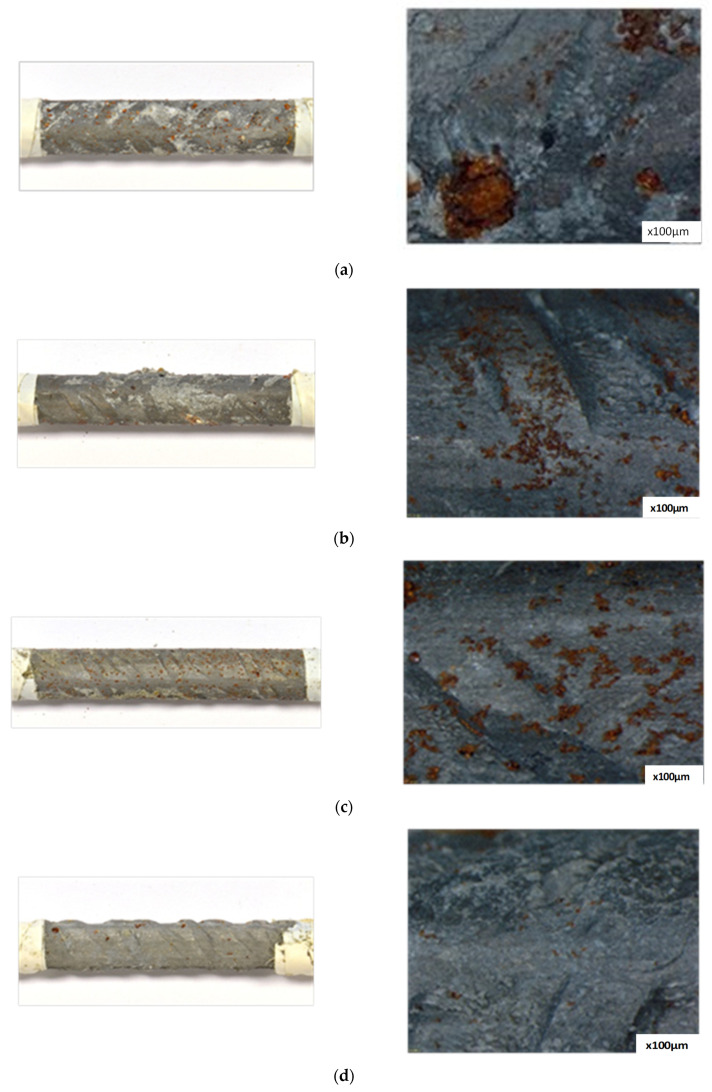

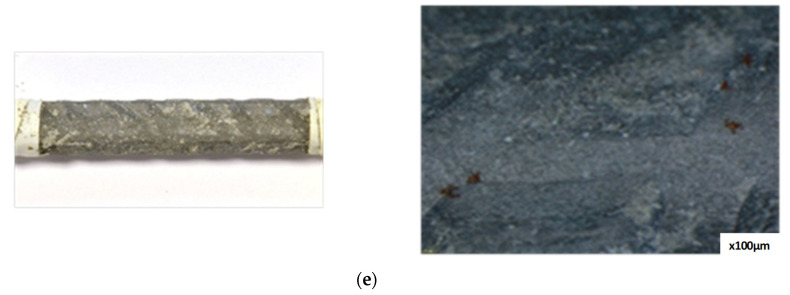
Visual and microscope examination of chloride-contaminated mortars, (**a**) reference; (**b**) 0.088% of AA; (**c**) 0.044% of AA; (**d**) 0.009% of AA; (**e**) 2.6% of NO_2_^−^.

Figure 7 shows the surface of the steel rebars after they were extracted from mortars at the end of 240 days of corrosion testing. Areas with localized pitting where the steel had been removed were observed in the steel of reference mortar. Shallow brown spots homogeneously distributed along the surface of the steel were observed for the reinforced rebar of mortars with high ascorbic acid content (0.088 and 0.044% of AA). These spots become more isolated in the surface of the steel with 0.009% of AA. The steel bar of the mortar with nitrite was covered with a colorless passive film with scarce of rust stains. Visual observation supports results from polarization resistance and impedance spectroscopy measurements.

### 3.3. Characterization Analysis in Cement Paste with Differential Thermal and Thermogravimetric Analysis (DTA-TG)

When the mortars with high content of ascorbic acid were manufactured, they presented a lumpy appearance, suggesting that there might be some interaction with the hydrated cement matrix. For this reason, a study of the influence of ascorbic acid on the hydrated cement paste was carried out. The cement pastes used in this study do not have any added chloride or nitrite ions.

**Figure 8:**
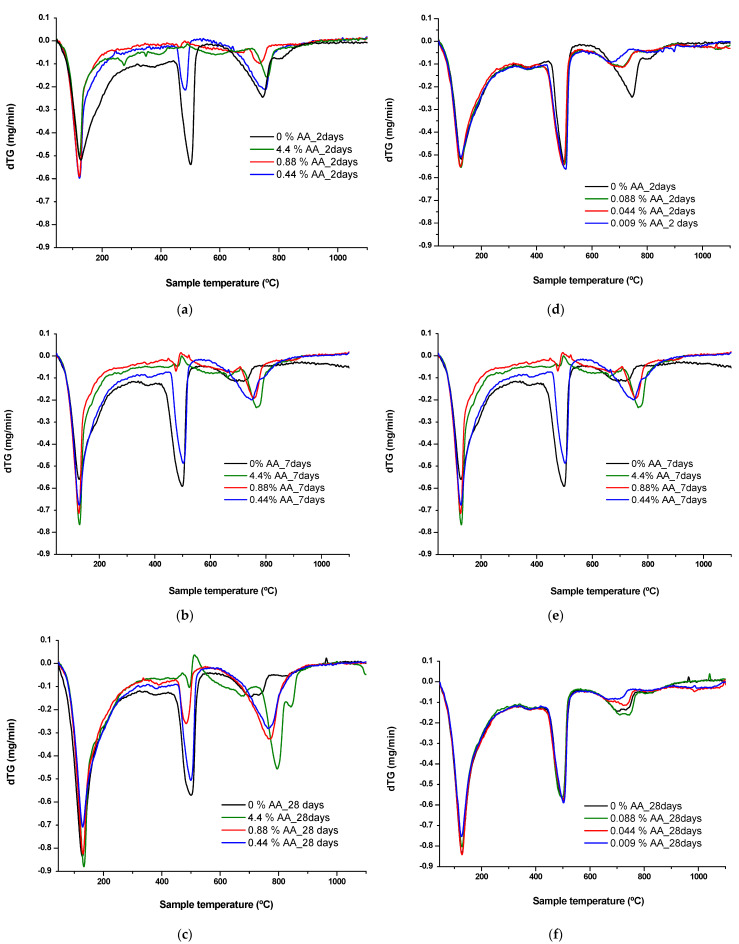
Thermal analysis (dTG vs. temperature) of cement pastes with 4.4, 0.88 and 0.44% of AA (**a**–**c**); 0.088, 0.044, 0.009% of AA (**d**–**f**); after 2, 7 and 28 days.

**Figure 9:**
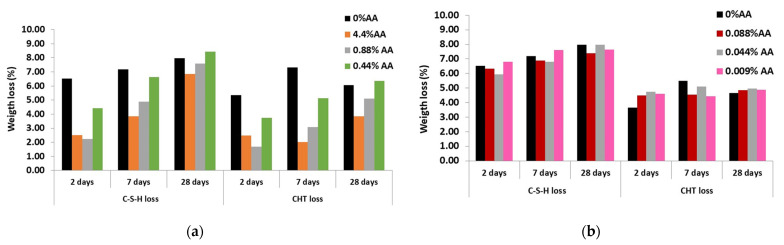
Weigth loss (wt.%) of cement pastes with AA (**a**) 4.4, 0.88 and 0.44% of AA; (**b**) 0.088, 0.044, 0.009% of AA.

The thermogravimetric derivative of the TG (dTG) versus temperature was represented since it allows a more accurate identification of the endothermic peaks of different hydrated cement compounds. Hydrated cement pastes made with addition of the highest amounts of ascorbic acid, 4.4, 0.88 and 0.44% of AA and a reference without ascorbic acid were tested at 2, 7 and 28 days and are shown in Figure 8a–c, respectively. The same figure also shows dTG graphs of the pastes with the lowest additions of ascorbic acid, 0.088, 0.044 and 0.009% of AA in Figure 8d–f, respectively. The first endothermic peak is normally associated with the dehydration of C-S-H gels (labeled as C-S-H loss) considered in the region between 140 °C [42] and the beginning of the portlandite dehydroxilation temperature. The portlandite decomposition (labeled as CHloss) occurs between 400 and 550 °C and corresponds to the second endothermic peak. The mass loss ranged from 550 to 1100 °C is generally associated with the weight loss of CO_2_ (labeled as CO_2_ loss) due to decomposition of calcium carbonate. Figure 9 provides the percentage of weight loss corresponding to these peaks. The CHloss also considers the water loss derived from the carbonated portlandite as CHTloss = CHloss + 0.41 CO_2_loss [43].

The peaks corresponding to the C-S-H gel and portlandite are reduced at early ages (two and seven days) when higher amounts of ascorbic acid (4.4, 0.88 and 0.44% of AA) were added, as shown in Figure 8a–c and Figure 9a. These data confirm that the addition of the high amounts of ascorbic acid (4.4 and 0.88 of AA) delays the hydration of C_3_S and C_2_S of cement paste, decreasing the formation of C-S-H gel and portlandite (CH) at early ages (two and seven days) and compromising the alkalinity of the matrix and its passivating effect against corrosion. After seven days, portlandite is detected in all the pastes made with inhibitor. Nevertheless, the higher the AA content, the smaller the quantity of portlandite.

However, the addition of lower amounts of ascorbic acid (0.088, 0.044 and 0.009% of AA) to the mixing water does not modify the hydration of cement, as shown in Figure 8d–f and Figure 9b. The weight loss of C-S-H gel and portlandite at 28 days was produced at a similar extent to the reference mortar and was not affected by the concentration of the inhibitor.

**Figure 10:**
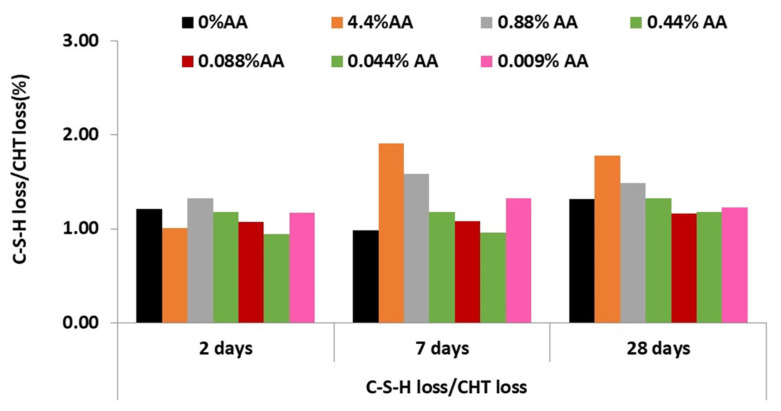
Gel C-S-H/Portlandite ratio (wt.%) of cement pastes with AA.

Figure 10 shows the C-S-H gel versus portlandite ratio, which would give an indication of the degree of hydration of the pastes.

With high inhibitor contents, although the formation of gel and portlandite is delayed at an early age, the gel/portlandite ratio remains constant. After seven days, the hydration increases, but the portlandite formation is lower than that of C-H-S gel, so the gel/portlandite ratio increases. The lower the inhibitor concentration, the more closely the matrix of the paste with inhibitor resembles the paste without inhibitor.

However, with the addition of lower amounts of ascorbic acid (0.088, 0.044 and 0.009% of AA), the C-S-H gel/portlandite ratio in the pastes with inhibitor remained similar at all ages to that of the paste without inhibitor.

### 3.4. pH Measurements

**Figure 11:**
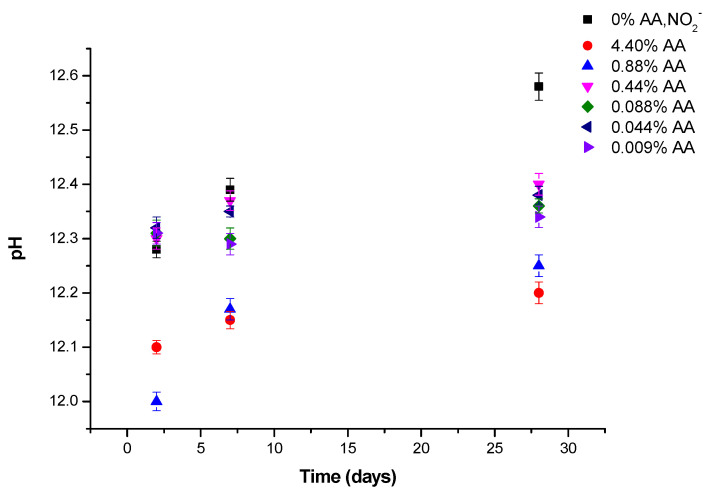
pH of cement pastes with different amounts of AA determined by ESL method.

The three most widely used destructive methods to determine the pH in cement pastes are the expression method [44], in situ leaching [45] and the ex situ leaching tech-nique (ESL) [46]. In the present study, the pH of cement pastes was determined using the ESL method. Figure 11 shows the effect of ascorbic acid addition on pH of cement pastes without chlorides measured at 2, 7 and 28 days. The results were the average of two pH measurements. The pastes with lower pH values are those with the higher ascorbic acid content (4.4 and 0.88% of AA). The rest of the pastes maintain pH values between 12.3 and 12.4 similar to that of reference paste up to 28 days when it reaches a pH value of 12.6. The pH values are consistent with the gel/portlandite ratios obtained at different ages. At early ages, the delayed hydration in the pastes with higher inhibitor content decreases the pH. As hydration evolves, the portlandite content is lower than the C-S-H gel content in the pastes with higher inhibitor content; therefore, their pH is also lower.

## 4. Discussion

The present work shows the performance of a green inhibitor, ascorbic acid, in comparison with a widely studied inorganic inhibitor, nitrite ions. The assessment comprises two aspects: the ability of ascorbic acid to inhibit corrosion and its interaction with the hydrated cement matrix. Corrosion studies were carried out on mortars, and the interaction with the matrix was evaluated on cement pastes. Inhibitors and chloride ions were added from the beginning to the mixing water. Electrochemical studies reveal that the inhibition ability of ascorbic acid is concentration-dependent. Ascorbic acid at concentrations above 10^−2^ mol/L (0.088% of AA in weight of cement) acts as a corrosion activator in mortars with 2% chloride ions (1.13 mol/L). However, below this concentration, its inhibitory efficacy increases as the inhibitor concentration decreases, reaching its maximum inhibitory power at a concentration of 10^−3^ mol/L of ascorbic acid, which is equivalent to 0.009% of AA in weight of cement. At this concentration, the inhibitory efficacy of ascorbic acid is similar to that of nitrite ions. Similar conclusions regarding the optimum concentration of ascorbic acid were obtained in electrochemical tests in solution and lower additions of chloride ions of 5 × 10^−3^ [21] and 0.14 mol/L [23] and sulphate ions (0.01 mol/L, pH = 4) [22] as depassivating ions.

A possible explanation for the effect of inhibitor concentration on the reduction in the corrosion rate lies in the ability of the ascorbic acid to form chelates with the transition metal ions through the O(3) and O(2)–H groups [47,48]. Two mixed valence Fe(II,III)–ascorbate complexes with bridging hydroxyl groups have been reported by Rao [49]. The solubility of the iron complexes is determined by the iron/ligand ratio, an increase in the ascorbic acid concentration and the resulting decrease in the iron/ligand ratio, providing soluble complexes, while low ascorbic acid addition leads to insoluble complex formation [50]. L-ascorbic acid exhibits a pro-oxidant activity by reducing Fe^3+^ to Fe^2+^ and producing dehydroascorbic acid and soluble Fe(II) chelates [51]. The high corrosion current density of concentrations above 0.01 mol/L of AA could be attributed to the formation of soluble Fe(II)–ascorbate. These complexes were identified via Mossbauer spectroscopy at the mild steel surface after exposure to ascorbate solution of concentrations higher than 0.05 mol/L [52]. The reduction in the Fe(III) layer could leave voids through which chloride ions enter and deteriorate the passive film [53]. Nevertheless, concentrations below 0.01 mol/L of AA ascorbic acid inhibit corrosion, possibly a consequence of the formation of stable complex Fe(III)–ascorbate chelate on the steel surface [54]. These insoluble Fe(III)/AA complexes may act as a physical barrier blocking the adsorption of Cl^−^ at the surface of passive film and restricting the diffusion of Fe(II) ions from the metal surface, reducing the corrosion rate [55,56].

The inhibitory efficacy of ascorbic acid at the lowest concentration 10^−3^ mol/L (0.009% of AA by weight of cement is similar to that of nitrite ions using a 1000 times higher concentration 1.13 mol/L of NO_2_^−^ ions (2.6% of NO_2_^−^ by weight of cement). The protective behavior of nitrites and ascorbic acid evaluated with linear polarization measurements can also be confirmed by impedance spectroscopy. The capacitive arcs with the significantly largest diameter at lower frequencies, the highest values of Rct (16,292 and 16,837 kΩ∙cm^2^) and minimum values of phase angle (−81° and −79°) corresponded both to the mortar with nitrite and ascorbic acid with lower content and suggested the formation of a comparable efficiency in the passive layer formation. This passive layer could be due to the adsorption of the Fe(III)–ascorbate complex on the protective layer of γ-FeOOH producing a barrier for further ferrous ion diffusion into the electrolyte [56,57]. The Rm resistance value was higher in mortars with ascorbic acid than the mortar without any inhibitor; however, values are quite similar for the three lower additions (9.11 to 9.59 KΩ∙cm^2^). This could be explained by the changes in the cement paste matrix due to precipitation of insoluble salts from the degradation products of ascorbic acid, such as 2,3-diketo-Lgulonic, resulting in a denser mortar [14,15,16,17,18].

The influence of ascorbic acid on the cement matrix is evaluated with thermogravimetric analysis and also depends on the concentration of the inhibitor. High amounts of ascorbic acid impede the hydration of the cement paste at early ages (2 days), decreasing both the formation of C-S-H gel and portlandite. From this age, a decrease in portlandite with respect to the C-S-H gel is observed, possibly due to the calcium complexation and neutralization reactions because of the acid characteristics of this compound.

Calcium and silicate complexing ability was detected in sugars. The ascorbic acid molecule is a derivative of glucose; therefore, its structure would also allow it to form calcium complexes. According to Thomas [58], the analysis of the aqueous phase of hydrating cement paste mixed with sugar solutions reveals increases in Ca^2+^, OH^−^ concentration, Si, Fe and Al associated with a calcium and silicate complexing ability of sugars. Similar findings were observed by Juenger and Jennings [59] who studied the influence of sugar addition on the hydration and microstructure of cement paste, concluding that sugar addition alters the rate of cement hydration and modifies the microstructure of calcium silicate hydrate (C-S-H). Furthermore, the presence of ester groups in the ascorbic acid molecule could inhibit the formation of the C-S-H gel and portlandite in the early ages, even modifying their morphology [60]. Chen et al. [61] confirmed the delaying mechanisms of ascorbic acid on C_3_A hydration in two ways: the adsorption of AA on the surface of the cement particle and AA-Ca^2+^ complexation. Ascorbic acid concentrations below 10^−2^ mol/L do not modify the hydration of cement paste at any of the ages studied.

The pH values, according to ESL method, obtained in cement pastes with amounts of ascorbic acid below 5 × 10^−2^ mol/L (0.44% of AA) were similar to those of the reference cement paste between two and seven days. At the age of 28 days, although the pH value of the pastes with ascorbic acid was lower than that of the reference paste, this does not translate into a dissolution of the portlandite to neutralize such a drop in pH as can be seen in Figure 9b. Conversely, the portlandite decreases in the paste with a higher amount of ascorbic acid as a result of AA-Ca^2+^ complexation and the neutralization reaction to compensate the lower pH of these cement pastes, as shown in Figure 9a.

In general, the pH measured with the ESL method in cement pastes is somewhat lower than that obtained by others authors. Deschner et al. studies [62] the concrete pore solutions were extracted using the expression method and the pH of the pore solutions were analyzed with a pH electrode, calibrated against KOH solutions. During the first eight hours the pH is measured was 13.1 ± 0.1 due to the fast dissolution of the alkalis sulphates and the presence of the calcium sulphate phases of cement. After that, the pH value was increased to 13.7 ± 0.1 at one day due to the consumption of calcium sulphate phases and the formation of portlandite. After one day, the hydroxide concentration and pH values are slightly increased over time due to continued hydration of the clinker phases. Sagues et al. [45] reported pH values of different concrete mixes between 12.4 and 12.8 a few hours after the start of the test and pH values between 12.8 and 13.4 after two weeks to one month with the in situ leaching method and reported that ex situ leaching underestimates hydroxide ion concentration likely because of the dilution effect. In general, the pH measured with the ESL method in cement pastes is somewhat lower than that obtained by other authors. Deschner et al. studied [62] the concrete pore solutions extracted using the expression method, and the pH of the pore solutions was analyzed with a pH electrode, calibrated against KOH solutions. During the first eight hours, the pH measured was 13.1 ± 0.1 due to the fast dissolution of the alkali sulphates and the presence of the calcium sulphate phases of cement. After that, the pH value increased to 13.7 ± 0.1 at one day due to the consumption of calcium sulphate phases and the formation of portlandite. After one day, the hydroxide concentration and pH values were slightly increased over time due to continued hydration of the clinker phases. Sagues et al. [45] reported pH values of different concrete mixes between 12.4 and 12.8 a few hours after the start of the test and pH values between 12.8 and 13.4 after two weeks to one month with the in situ leaching method and reported that ex situ leaching underestimates hydroxide ion concentration, likely because of the dilution effect.

## 5. Conclusions

The recent research mainly focused on the study of the inhibitor effect of L-ascorbic acid to reduce chloride-induced corrosion in mortars. The inhibitory capacity of this eco-friendly compound was compared with the well-known inhibitory capacity of sodium nitrite. The influence of the additive in the cement paste was also evaluated. This study yields the following conclusions:

1. Ascorbic acid can be considered as a corrosion inhibitor in chloride-contaminated mortars. The inhibitory ability of ascorbic acid is sustained long-term (8 months). Its effectiveness is dependent on the inhibitor concentration added. Greater concentrations than 10^−2^ mol/L (0.088% of AA by weigh of cement) can give rise to the opposite effect, that is, increase the corrosion of reinforced steel, whereas equal or smaller concentrations inhibit the corrosion of chloride ions.
2. The highest efficiency of ascorbic acid as a corrosion inhibitor is reached with the addition of a concentration of 10^−3^ mol/L (0.009% of AA by weight of cement) in the mixing water (IE = 97.3%) and is similar to the nitrite addition with a concentration of 1.13 mol/L of NO^2−^ (2.6% NO^2−^), IE = 97.8%. The chloride ion/ascorbic acid molar ratio (Cl^−^/(AA) = 1129) for this concentration of ascorbic acid is 1000 times higher than the chloride-nitrite molar ratio (Cl^−^)/(NO^2−^) = 1.
3. An ascorbic acid concentration below 10^−2^ mol/L (0.088% of AA by weight of cement) does not modify the hydration or the pH of the cement matrix.

## Figures and Tables

**Table 1:** Chemical composition of the cement (%).

SiO_2_	Al_2_O_3_	Fe_2_O_3_	CaO	MgO	SO_3_	K_2_O	Na_2_O	TiO_2_	P_2_O_5_	Cl^−^	LOI
20.7	3.5	4.5	64.3	1.05	2.48	0.53	0.36	0.28	0.13	0.04	1.7

**Table 2:** Ascorbic acid dosage in mortar specimens and cement pastes.

Ascorbic Acid							
mol/L	-----	0.5	0.1	0.05	0.01	0.005	0.001
(% by weight of cement)	-----	4.403	0.881	0.440	0.088	0.044	0.009
[Cl^−^]/[AA]	-----	2.26	11.28	22.57	112.83	225.65	1128.65

**Table 3:** Inhibitor efficiency of 2% chloride-contaminated mortars.

Inhibitor Content	Efficiency (%)
0.088% AA	78.5
0.044% AA	87.4
0.009% AA	97.3
2.6% NO_2_^−^	97.8

**Table 4:** Fitting impedance data using the EEC on Figure 5 for steel bars embedded in chloride-contaminated mortars, with 0.088, 0.044 and 0.009% of AA and 2.6% of NO_2_^−^.

	R_s_ + R_m_ (KΩ∙cm^2^)	CPE_m_		R_ct_ (KΩ∙cm^2^)	CPE_dl_	
Mortar Denomination		Y_0m_ (Ω^−1^·cm^−2^·s^n^)	n_1_		Y_0dl_ (Ω^−1^·cm^−2^·s^n^)	n_2_
0%NO_2_^−^, AA	4.95	3.57 × 10^−3^	0.22	943.55	5.83 × 10^−2^	0.84
0.088%AA	9.11	4.90 × 10^−3^	0.13	1146.95	1.01 × 10^−2^	0.89
0.044%AA	9.32	2.94 × 10^−3^	0.16	1440.75	7.20 × 10^−3^	0.80
0.009%AA	9.59	2.88 × 10^−3^	0.20	16,837.00	1.50 × 10^−3^	0.90
2.6%NO_2_^−^	4.38	2.51 × 10^−3^	0.22	16,291.78	2.09 × 10^−3^	0.93

## Data Availability

Not applicable.
